# Supplementary figures and images for: Active poroelastic two-phase model for the motion of physarum microplasmodia
Source: PLoS One. 2019 Aug 9;14(8):e0217447. doi: 10.1371/journal.pone.0217447 (PMC6688797; doi:10.1371/journal.pone.0217447)

Body reference frame

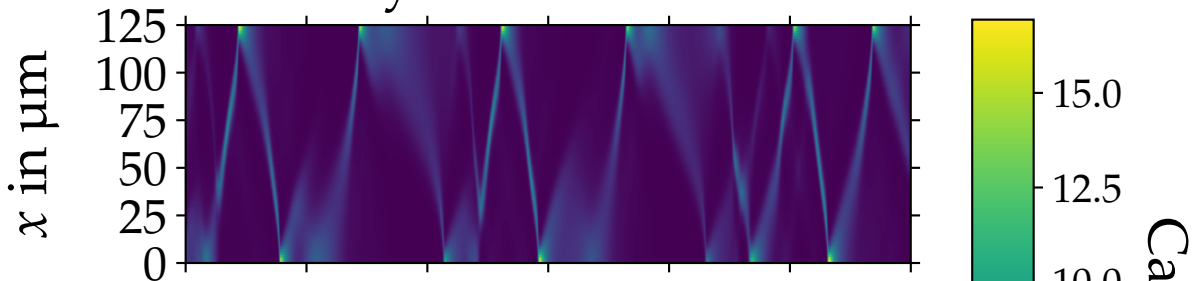

Lab frame

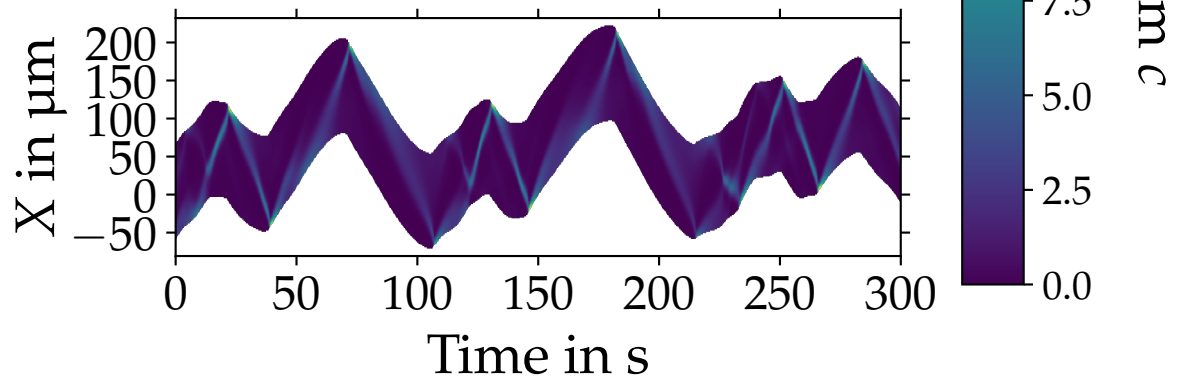

Supplement: S1 Fig — We solve our model equations in the gel’s body reference frame and the resulting quantities are defined in this frame. However, observers are located in the lab frame. The quantity’s transformation from body reference to lab frame is given by the displacement field u with X0 = x0 + u(x0). Here, x0 the position in the body reference and X0 is the position in the lab frame. Parameters from Fig 2 (left) in the main text. (PDF) [file pone.0217447.s001.pdf]

Body reference frame

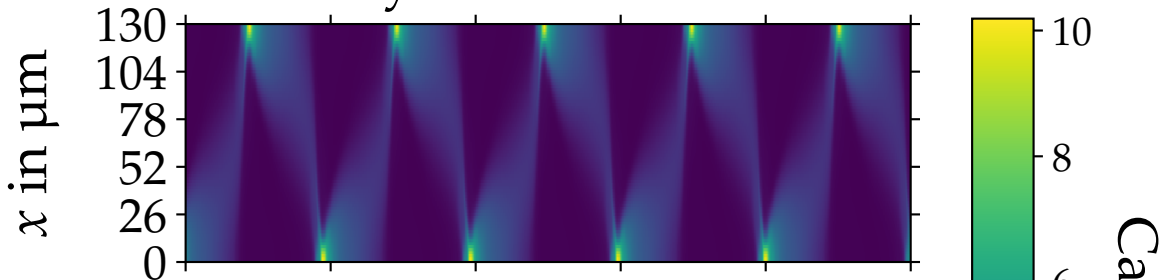

Lab frame

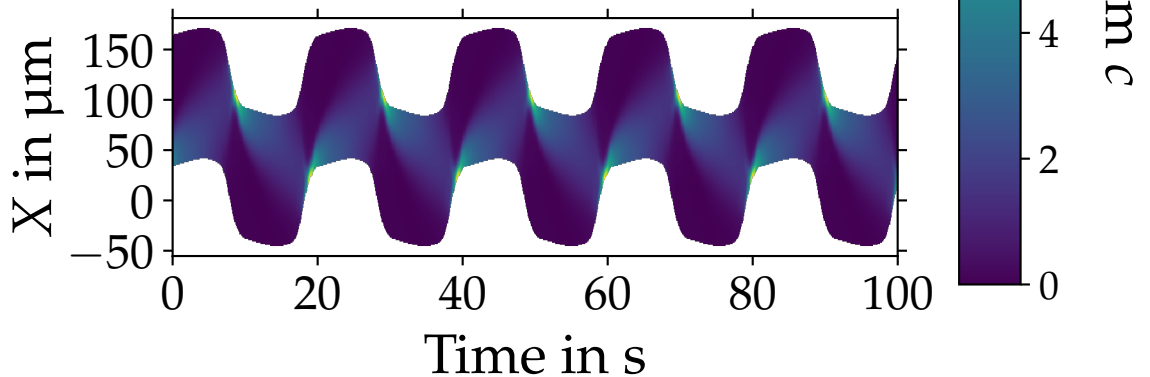

Supplement: S2 Fig — Parameters from Fig 2 (right) in the main text. (PDF) [file pone.0217447.s002.pdf]

Body reference frame

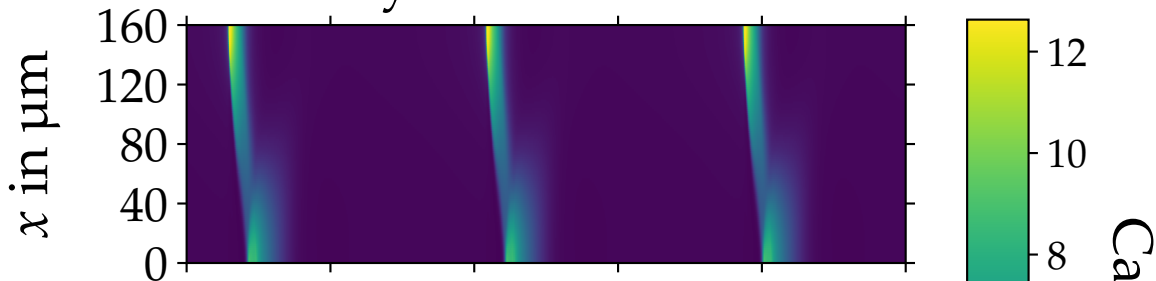

Lab frame

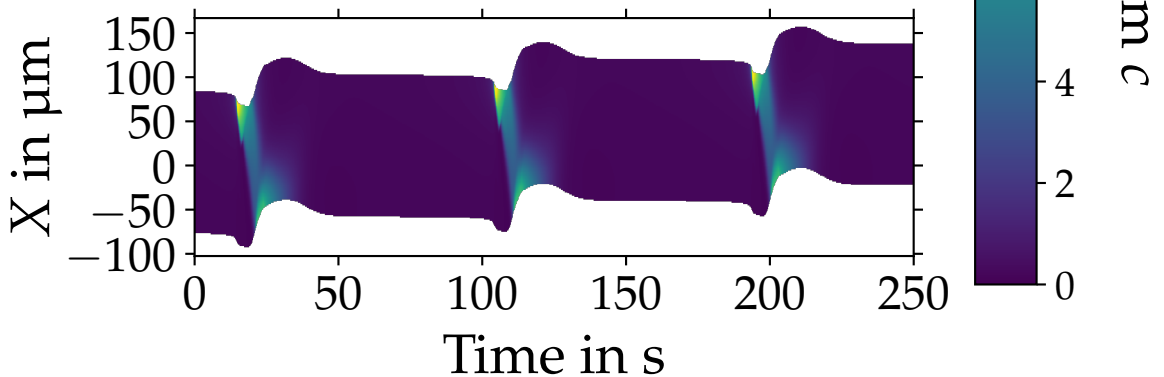

Supplement: S3 Fig — Parameters from Fig 3 in the main text. (PDF) [file pone.0217447.s003.pdf]

Body reference frame

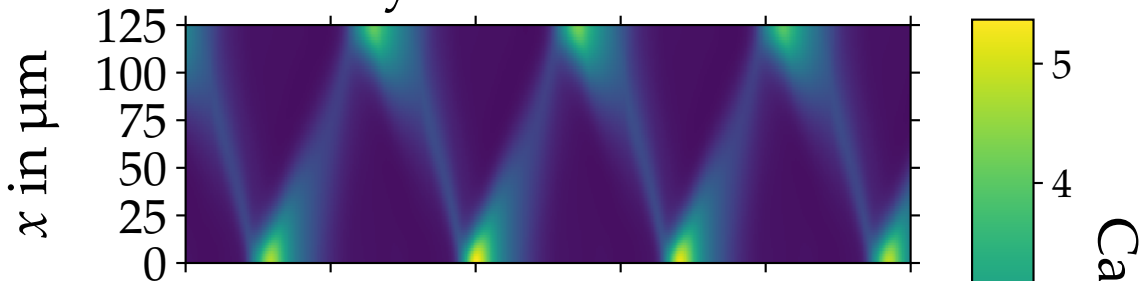

Lab frame

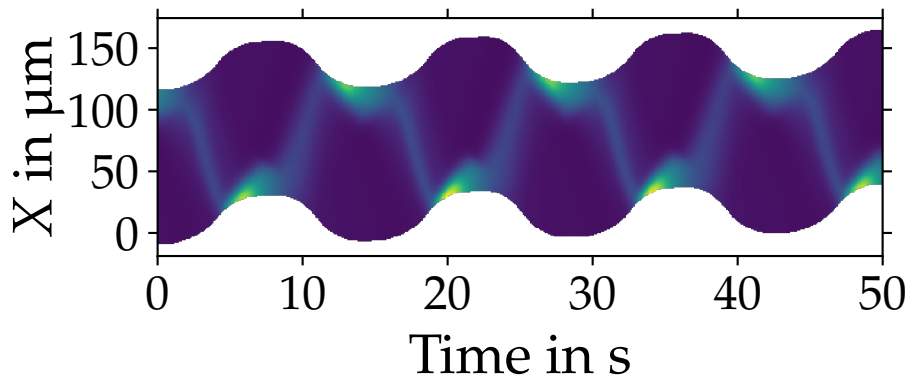

Supplement: S4 Fig — Parameters from Fig 4 in the main text. (PDF) [file pone.0217447.s004.pdf]

Net speed in  $\frac{\mu\text{m}}{\text{s}}$

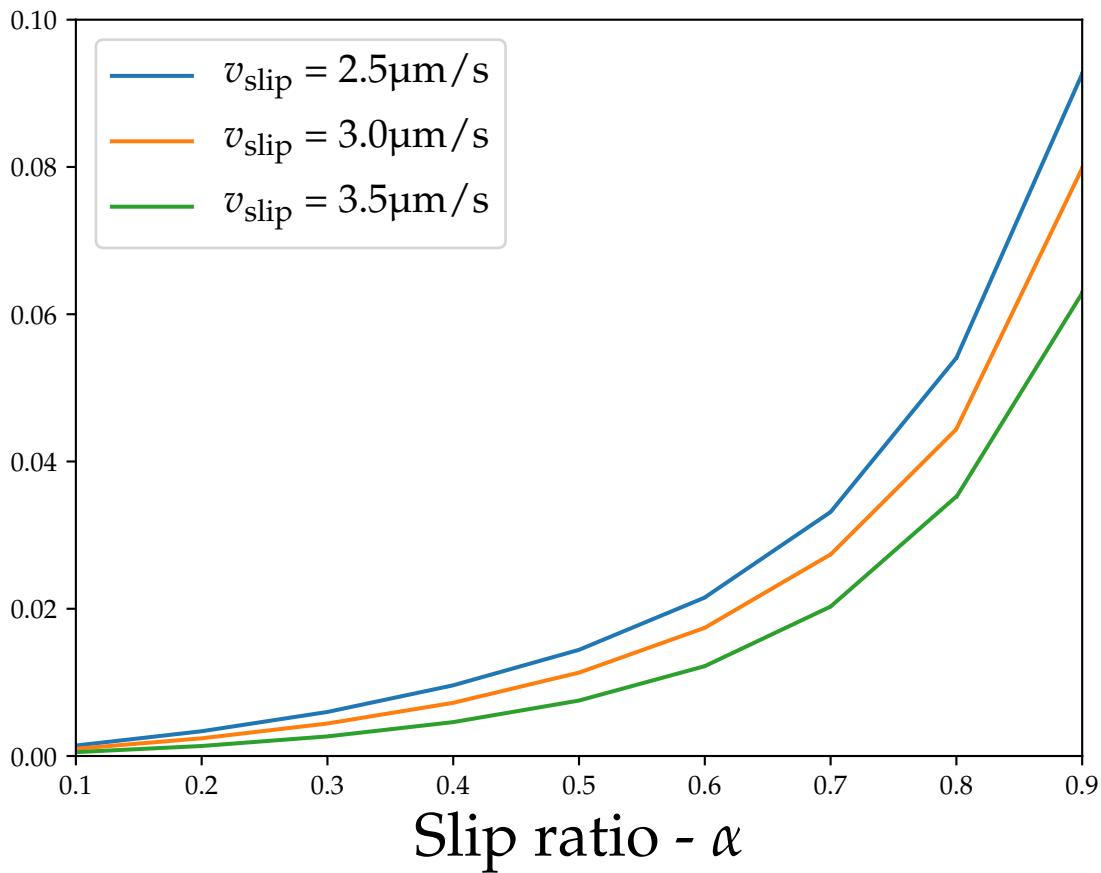

Supplement: S5 Fig — Parameters B = 3.5, ψ = 0.1/s, L = 125 μm and F = 12.3. (PDF) [file pone.0217447.s005.pdf]

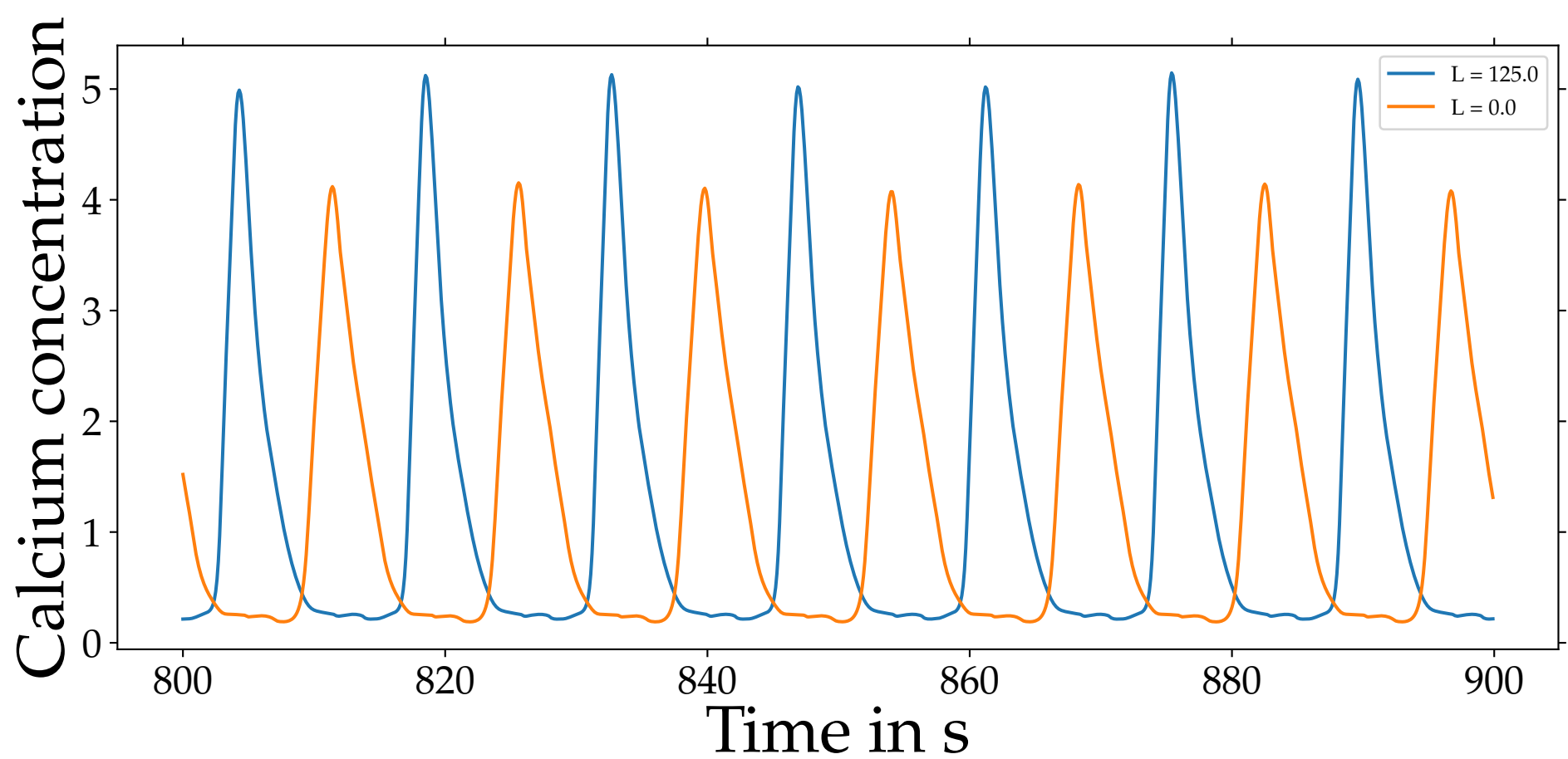

Supplement: S6 Fig — The magnitude of emerging waves is always higher at the MPboundariess front and it is moving into this direction. Parameters from Fig 4 in the main text. (PDF) [file pone.0217447.s006.pdf]

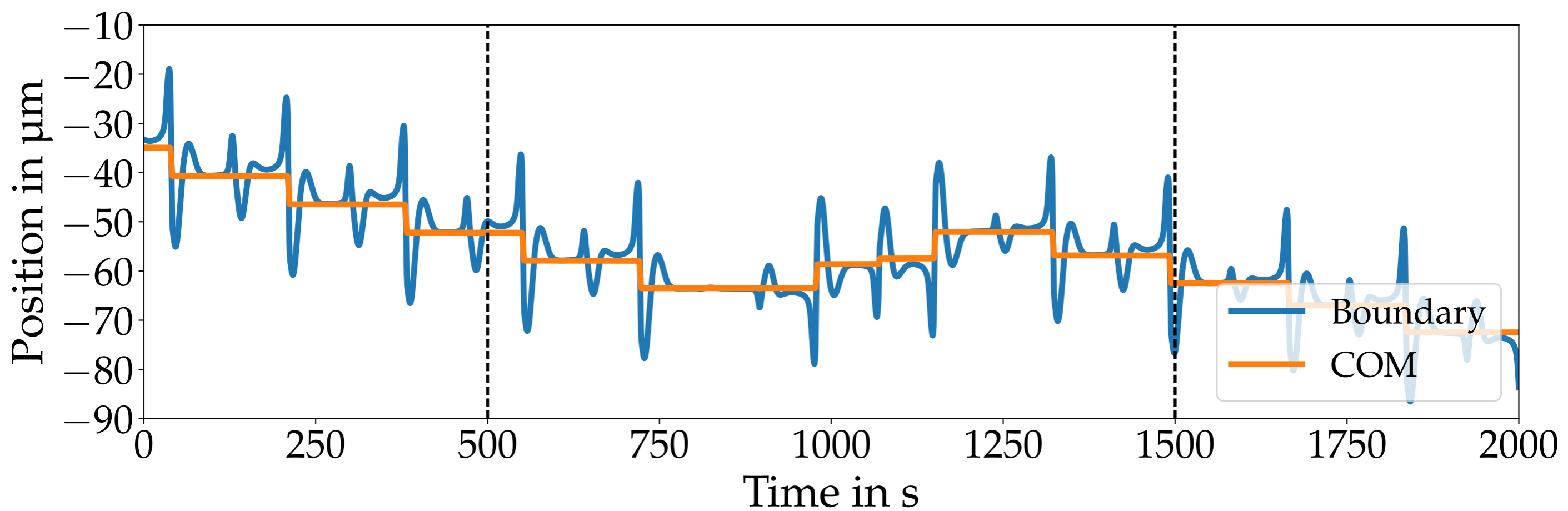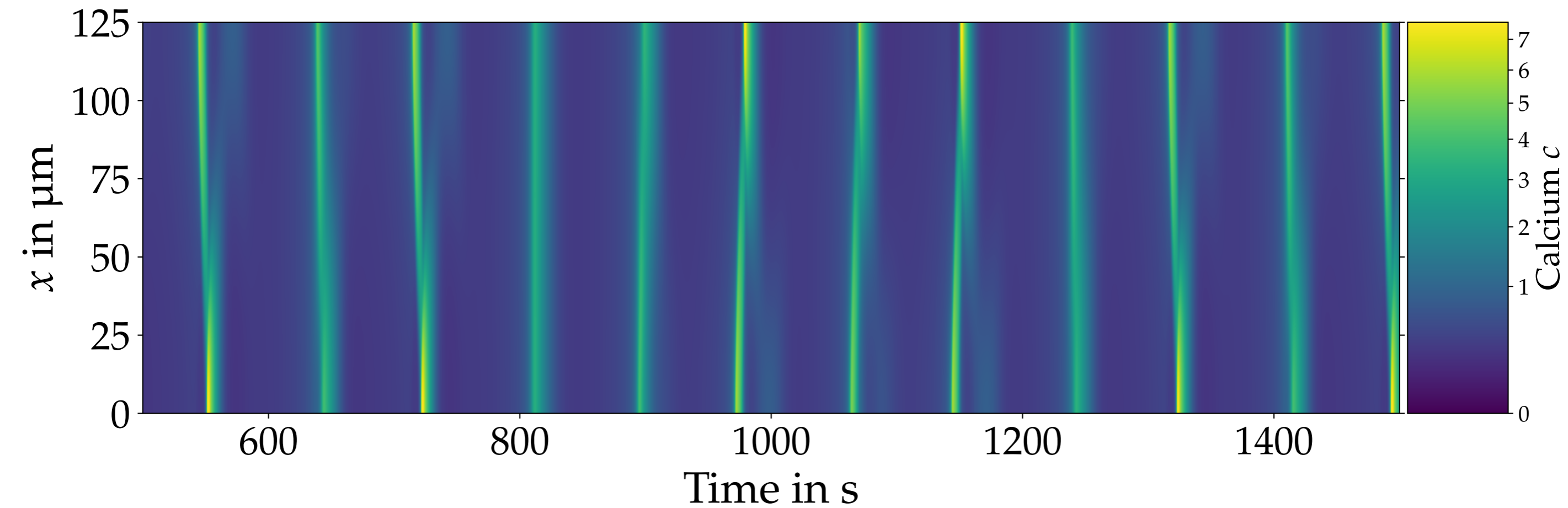

Supplement: S7 Fig — Parameters: B = 2.5, α = 0.1, vslip = 3.1, γ0 = 10−5 kg/s, L = 125 μm and F = 15.4. (PDF) [file pone.0217447.s007.pdf]
