# Supplementary material for: Active poroelastic two-phase model for the motion of physarum microplasmodia
Source: PLoS One. 2019 Aug 9;14(8):e0217447. doi: 10.1371/journal.pone.0217447 (PMC6688797; doi:10.1371/journal.pone.0217447)
Supplement: S1 Text — provides the following: i) a visual comparison of a moving MP in lab and body reference frame ii) Dispersion relations for the linear stability analysis iii) A table with the default parameters. (PDF) [file pone.0217447.s008.pdf]

Supplementary Information: Active poroelastic Two-Phase Model for the Motion of Physarum Microplasmodia

Dirk Alexander Kulawiak Jakob Löber Markus Bär
Harald Engel

1 Supplementing figures

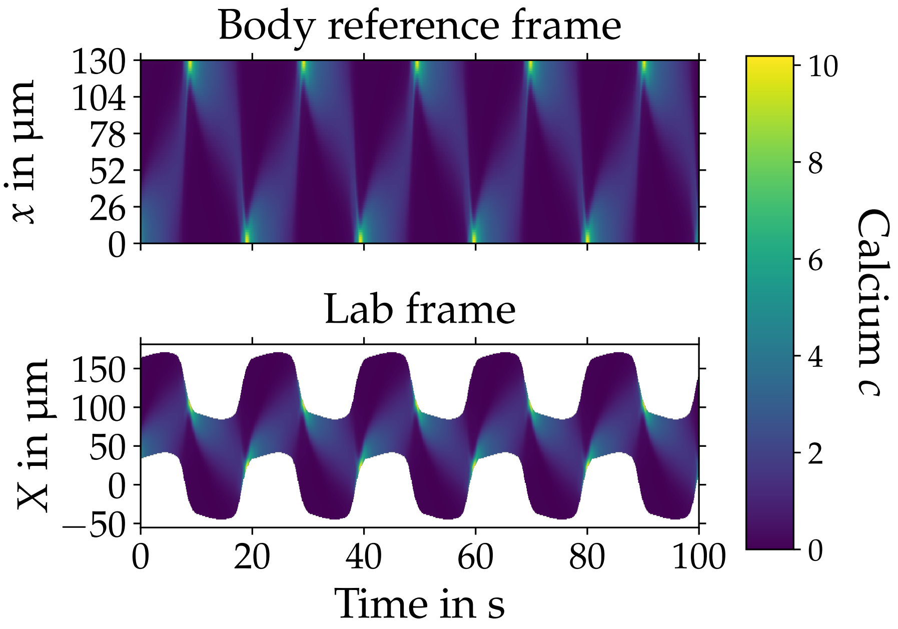

Figure S1: **Regular motion with nonlinear substrate friction without calcium kinetics in in body reference (top) and lab frame (bottom).** We solve our model equations in the gel's body reference frame and the resulting quantities are defined in this frame. However, observers are located in the lab frame. The quantity's transformation from body reference to lab frame is given by the displacement field u with $X_0 = x_0 + u(x_0)$. Here, x_0 the position in the body reference and X_0 is the position in the lab frame. Parameters from Fig. 2 (left) in the main text.

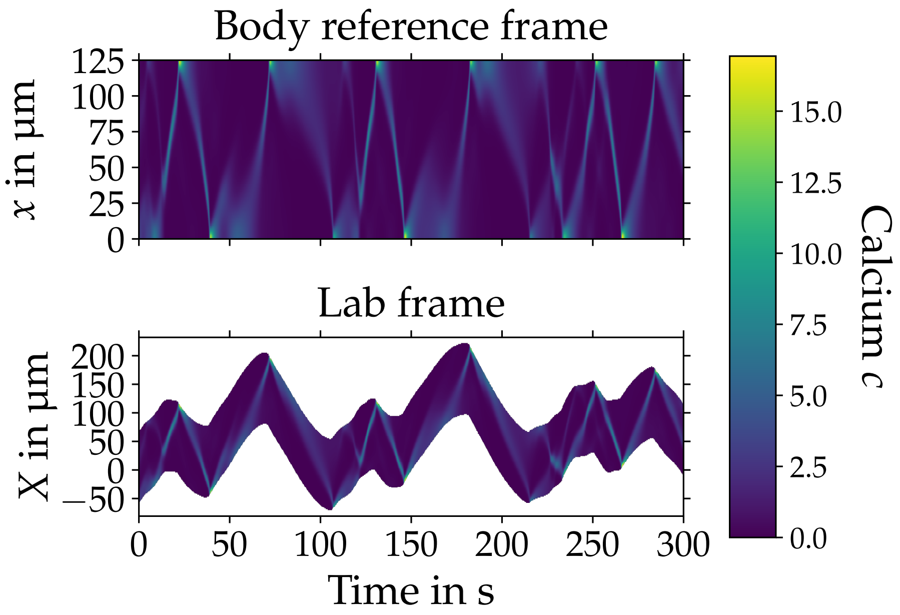

Figure S2: **Irregular motion with nonlinear substrate friction without calcium kinetics in in body reference (top) and lab frame (bottom).** Parameters from Fig. 2 (right) in the main text.

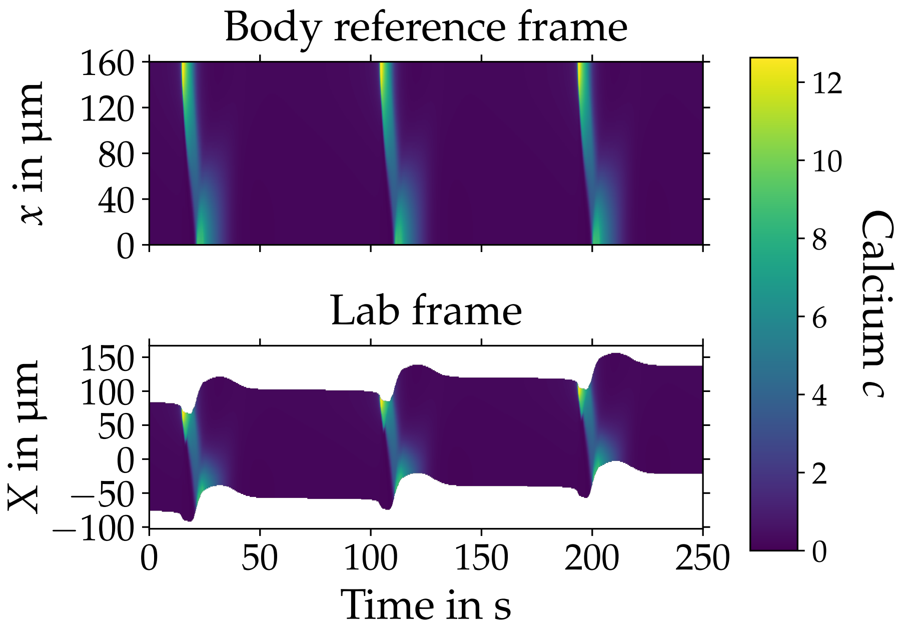

Figure S3: **Moving type 1 MP in body reference (top) and lab frame (bottom).** Parameters from Fig. 3 in the main text.

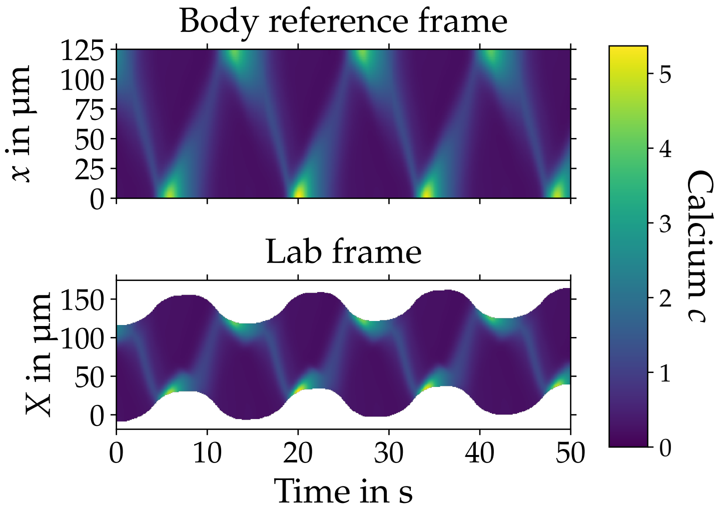

Figure S4: **Moving type 2 MP in body reference (top) and lab frame (bottom).** Parameters from Fig. 4 in the main text.

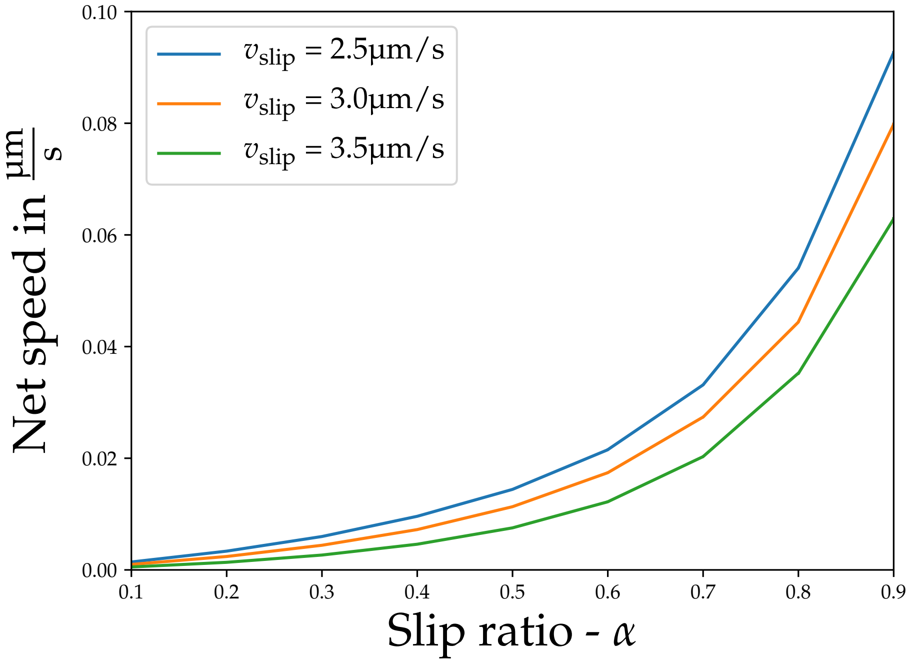

Figure S5: **MP net speed increases with a larger slip ratio α.** Parameters $B = 3.5$, $\psi = 0.1\,\mathrm{s}^{-1}$, $L = 125\,\mu\mathrm{m}$ and $F = 12.3$.

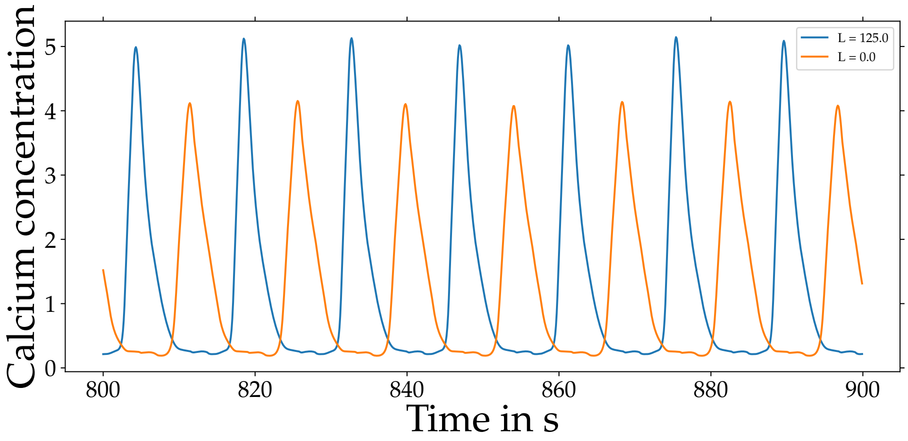

Figure S6: **Calcium concentration *c* at the the MP's boundaries**. The magnitude of emerging waves is always higher at the MP's front and it is moving into this direction. Parameters from Fig. 4 in the main text.

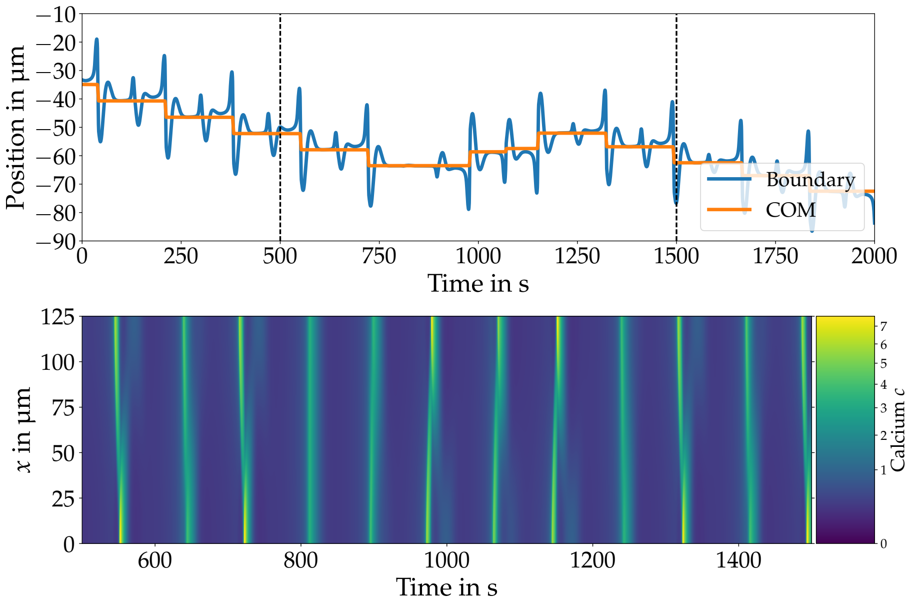

Figure S7: **COM (orange) and boundary (blue) trajectories (top) and calcium dynamics (bottom) with irregularly switching polarity**. Parameters: $B = 2.5$, $\alpha = 0.1$, $v_{\mathrm{slip}} = 3.1$, $\gamma_0 = 10^{-5}\,\mathrm{kg/s}$, $L = 125\,\mu\mathrm{m}$ and $F = 15.4$.

2 Parameters

Table S1: **Physarum parameters**

Par	Description	Value	Units
Δt	Numerical time step	0.005	s
D_c	Calcium diffusion coefficient	200	$\mu m^2 \, s^{-1}$
D_a	a diffusion coefficient	5.	$\mu m^2 \, s^{-1}$
A	Reaction parameter	0.8	-
ψ	Temporal scale	0.105	s^{-1}
ρ_g	Gel fraction	0.25	-
ρ_f	Fluid fraction	0.75	-
η_g	Viscosity gel	$1.3 \times^{-4}$	$\frac{kg}{\mu m \, s}$
η_f	Viscosity fluid	2×10^{-9}	$\frac{kg}{\mu m \, s}$
β	Friction between both phases	2×10^{-7}	$\frac{kg}{\mu m^3 \, s}$
E	Young modulus	0.001	$\frac{kg}{\mu m \, s^2}$

These parameters are used throughout this work and any derivation is explicitly marked. Taken from Ref. [40] in the main text.
