# Supplementary material for: Active poroelastic two-phase model for the motion of physarum microplasmodia
Source: PLoS One. 2019 Aug 9;14(8):e0217447. doi: 10.1371/journal.pone.0217447 (PMC6688797; doi:10.1371/journal.pone.0217447)
Supplement: S1 Table — Taken from [40]. (PDF) [file pone.0217447.s009.pdf]

Table S1: **Physarum parameters**

Par	Description	Value	Units
Δt	Numerical time step	0.005	s
D_c	Calcium diffusion coefficient	200	$\mu m^2\,s^{-1}$
D_a	a diffusion coefficient	5.	$\mu m^2\,s^{-1}$
A	Reaction parameter	0.8	-
ψ	Temporal scale	0.105	s^{-1}
ρ_g	Gel fraction	0.25	-
ρ_f	Fluid fraction	0.75	-
η_g	Viscosity gel	$1.3\times^{-4}$	$\frac{kg}{\mu m\,s}$
η_f	Viscosity fluid	2×10^{-9}	$\frac{kg}{\mu m\,s}$
β	Friction between both phases	2×10^{-7}	$\frac{kg}{\mu m^3\,s}$
E	Young modulus	0.001	$\frac{kg}{\mu m\,s^2}$

These parameters are used throughout this work and any derivation is explicitly marked. Taken from Ref. [40] in the main text.
